# Association between Childhood Exposure to Family Violence and Telomere Length: A Meta-Analysis

**DOI:** 10.3390/ijerph191912151

**Published:** 2022-09-26

**Authors:** Xiao Yan Chen, Camilla K. M. Lo, Ko Ling Chan, Wing Cheong Leung, Patrick Ip

**Affiliations:** 1 Department of Applied Social Sciences, The Hong Kong Polytechnic University, Hung Hom, Hong Kong; 2 Department of Obstetrics & Gynaecology, Kwong Wah Hospital, Kowloon, Hong Kong; 3 Department of Paediatrics and Adolescent Medicine, The University of Hong Kong, Pokfulam, Hong Kong

**Keywords:** childhood exposure to family violence, child exposure to intimate partner violence, child maltreatment, telomere length, meta-analysis

## Abstract

The aims of this meta-analysis were to examine the association between childhood exposure to family violence and telomere length and the moderating variables that influence this association. Relevant works published on or before 1st September 2022 were identified through a search in five major databases in English and 19 articles (N = 18,977) finally met the inclusion criteria. A meta-analysis was conducted to compute the pooled effect size (correlation; *r*), and moderator analyses were performed using a random effects meta-analytic model. The studies yielded a significant inverse association between childhood exposure to family violence and telomere length, with a small effect size (*r* = −0.038, 95% CI [−0.070, −0.005], *p* = 0.025). Furthermore, the strength of this association was stronger in studies examining the co-occurrence of multiple types of violence than in those examining just one type (Q = 8.143, *p* = 0.004). These findings suggested that victims’ telomere length may be negatively influenced by childhood exposure to family violence and that such impairment appears to be stronger for those who are exposed to multiple types of violence. Future studies are necessary to examine the moderating and mediating factors underlying the association between childhood exposure to family violence and telomere length.

## 1. Introduction

Exposure to family violence during childhood is a significant public health and social welfare concern. Violence does not always involve tangible types, which is defined as the intentional use of physical force or power, threatened or actual, against others that may potentially result in injury, death, psychological harm, maldevelopment or deprivation [1]. The two most common types of childhood exposure to family violence are child maltreatment and child exposure to intimate partner violence [IPV] [2,3]. These two kinds of violence are highly prevalent worldwide. A meta-analytic study found the prevalence of child maltreatment to be 0.13 for sexual abuse, 0.23 for physical abuse, 0.36 for emotional abuse, 0.16 for physical neglect, and 0.18 for emotional neglect [4]. In the same vein, 0.06 of children were exposed to IPV in the past year [5]. It is widely accepted that childhood exposure to family violence exerts adverse effects on victims’ subsequent health outcomes, such as by increasing their likelihood of having physical health and psychiatric disorders [6]. However, the mechanism underlying the relationship between childhood exposure to family violence and health consequences has not been fully elucidated.

Intriguingly, the growing field of research on biological markers, such as telomere length and telomere shortening, has opened a unique avenue for understanding the deleterious physical and mental effects of childhood exposure to family violence [7]. Telomeres are nucleoprotein complexes, which comprise repetitive DNA sequences (in humans and other vertebrates, their nucleotide sequence is TTAGGG). Telomeres cap the ends of eukaryotic chromosomes, protecting genomic integrity from deterioration caused by replication flaws [8]. Telomeres become progressively shorter over time, primarily because of cell replication and oxidative stress, and cells enter a state of senescence when telomeres reach a critically short length [9]. Shortening of telomere length (TL) is related to early mortality [10] and to psychiatric illnesses such as anxiety disorders [11].

Several meta-analyses and systematic reviews have summarized extant evidence for the influence of adversity on TL. Systematic reviews have reported inverse relationships between TL and adverse childhood experiences, such as child maltreatment and loss of a close family member [12,13,14,15], experiences of threat-related early-life adversity [16], and chronic social stress, including extreme poverty and family disruption [17,18,19]. Furthermore, meta-analyses revealed that early life adversity, in the forms of adverse social environment and child maltreatment [7,20], along with exposure to stress and adversity, such as psychiatric illnesses and physical diseases [21], were significantly related to TL. Another meta-analysis found a significant relationship between childhood separation and telomere erosion, but not between telomere erosion and physical abuse, sexual abuse, or loss of a parent [22]. Whereas these studies have provided important evidence supporting the idea that exposure to life adversity is associated with TL, most studies have focused on a general and broad definition of adversity in which childhood exposure to family violence was included with other adversities, making it difficult to tease out the specific impact of childhood exposure to family violence on TL. As Pepper and colleagues (2018) pointed out that integrating the consequences of different kinds of stressors may explain the weak and variable association between exposures and TL [21], thus separating the relationships between different exposures and TL may help us know more about the association between the particular exposure and TL. This view echoes our current objective that specifically focuses on the relation between the high prevalence of family violence happened in childhood and TL.

Previous studies focusing on the association between childhood exposure to family violence and TL have had mixed findings. A longitudinal study of 236 children found that exposure to two or more kinds of violence (e.g., domestic violence, physical maltreatment) was significantly associated with accelerated telomere erosion during a 5-year period [23]. However, Ridout and colleagues (2019) found a positive association between childhood maltreatment and TL in a sample of 256 children [24]. Similarly, Küffer and colleagues (2016) found that a higher level of childhood maltreatment was marginally associated with longer buccal cell telomere length in a sample of 120 former Swiss child laborers and healthy controls [25]. Such conflicting findings fall short in advancing our understanding of the influence of childhood exposure to family violence on TL. A meta-analytic review of this correlation between the two is needed to provide a synthesis of findings that contribute to the understanding of biological changes related to childhood exposure to family violence. Such a deeper understanding could guide early intervention and prevention strategies to identify novel targets to help victims recover from childhood exposure to family violence, which may potentially ameliorate the acceleration of TL.

Many possible factors could explain the previous heterogeneity of findings on the association between childhood exposure to family violence and TL. For example, the extant studies differed in the length of time between exposure to adversity and TL assessment. Furthermore, many of them measured TL in adults [26,27], while others were conducted on children [23]. Most studies employed qPCR to assess TL [28,29], but some used other techniques [30]. The majority of extant studies collected data from both male and female participants [31,32], but others included only female participants [33,34]. Therefore, findings on the association between childhood exposure to family violence and TL could have been affected by any of the abovementioned factors.

The objectives of this meta-analysis were to quantitatively summarize the association between childhood exposure to family violence and TL and to explore how the reported strength of that association was affected by moderators.

## 2. Materials and Methods

### 2.1. Search Strategy

Study selection was carried out in line with items for systematic reviews and meta-analyses (PRISMA) checklist. A description of the systematic review criteria is detailed below.

We searched five major electronic databases (PubMed, Web of Science, PsycINFO, Scopus, and Medline) to identify studies published in English on or before 1st September 2022. Publications were systematically searched by their titles, keywords, and abstracts, and using the following three groups of keywords: (1) violence, victim, abuse, maltreat, neglect, trauma, adversity, adverse; (2) telomere, biomarker; and (3) infant, child, adolescent, newborn, youth. Additional relevant publications were identified by manually searching the reference lists of all of the retrieved articles. No additional articles were identified.

We employed EndNote bibliographic management software to organize the studies. Of 21,867 titles, we first removed 7540 duplicates. Then, we screened the titles of the remaining 14,327 articles and eliminated 14,242 articles. After that, we examined the full text of the remaining 85 articles and 19 of them were included in this meta-analysis.

### 2.2. Study Eligibility

Studies were included if they met the following criteria: (1) they were written in English; (2) they provided sufficient data to calculate effect sizes on the relationship between childhood exposure to family violence and TL in human subjects. Studies were excluded if they did not include an analysis of primary data (e.g., if they were reviews, nonempirical, etc.).

### 2.3. Data Extraction and Quality Assessment

Data were extracted from all eligible studies by using a structured coding sheet that evaluated the following aspects: (1) publication information, including author(s), publication year, and country; (2) study characteristics, including sampling method, study design (cross-sectional or longitudinal design), and sample sizes; (3) participants’ demographic characteristics, including age, gender, and educational level; (4) violence-related information, including types of violence, the measurement types (e.g., self-report or by others), and time frame of violence; (5) TL-related information, including TL cell type (e.g., blood), the time frame of TL measurement (e.g., adulthood or childhood), and TL assay type (e.g., qPCR).

Each included study was evaluated for quality, using a quality assessment checklist. This checklist was adapted from a previous study [35]. Eight items such as sample characteristics were covered (see Table S1). Each item was evaluated as No (0) or Yes (1). Therefore, quality assessment scores ranged from 0 to 8, with the higher scores indicating higher study quality. Two independent raters evaluated and scored the studies independently, based on the checklist. We calculated the intraclass correlation coefficient (ICC) to assess interrater reliability. In this study, the quality assessment scores ranged from 7 to 8, and the interrater agreement for all of the included articles was at a high level (ICC = 0.91). Disagreements were resolved through discussion.

### 2.4. Statistical Analysis

Comprehensive Meta-Analysis (CMA) software version 3.0 (Biostat Inc., Englewood, NJ, USA) was used to conduct all statistical analyses. First, pooled correlations were used to examine the association between childhood exposure to family violence and TL. Data were derived from raw scores, for example, correlations, standard mean differences, and independent groups’ t-values. For studies with multiple correlation coefficients for the same variable, we averaged the multiple correlation coefficients, such that each study only involved one effect size for the final analysis. This method had been employed in a previous study [36]. We constructed a forest plot to demonstrate the correlation with 95% confidence intervals (CIs) in each study. In a fixed-effects model, studies are weighted according to their sample sizes, which has the limitation that it assumes a normal and homogenous distribution of the effect sizes. A random-effects model could take between-study and within-study variabilities into account, with that model being able to provide a more conservative estimate [37]. Therefore, this random-effects model is more appropriate for the current study as the articles we included came from different countries and had different sample sizes. We used Q statistics to test the heterogeneity between the included studies and subgroups, and I^2^ statistics to calculate the proportions of observed variance of the included studies. Values of I^2^ up to 25% were considered to be low amounts of heterogeneity, from 25% to 50% were moderate, and from 50% to 75% were high [38].

Next, we performed subgroup analyses to explore the sources of heterogeneity. The studies were divided into subgroups according to gender (both genders, or females only), whether the violence was co-occurring, the types of measurement for the violence (self-report or by others), source of tissue (blood, buccal swabs, or saliva), telomere measurement technique (qPCR or other techniques), timing of measuring telomeres (childhood or adulthood), whether the covariates were controlled, and sample sizes (small, medium, and large). Specifically, in the current study, the definition of co-occurrence of family violence (family polyvictimization) is two or more types of family violence rather than repeatedly occurring episodes of one single type of family violence [39,40]. A sensitivity analysis was performed using “one-study-removed”. We showed the result if removal of a study affected the association.

Finally, publication bias was visually examined by using a funnel plot delineating individual studies’ effect size against the standard error of the effect size and quantitatively tested by Egger’s regression and Begg–Mazumdar rank correlation [41,42]. The statistical significance of the publication bias was presented when the *p*-value was less than 0.05. If there was publication bias, the trim and fill algorithm, a compensation technique for publication bias, was then used to impute the effect size estimates for missing studies in order to obtain an unbiased effect size, which was then compared with the original effect size [43].

## 3. Results

### 3.1. Study Characteristics

**Figure 1:**
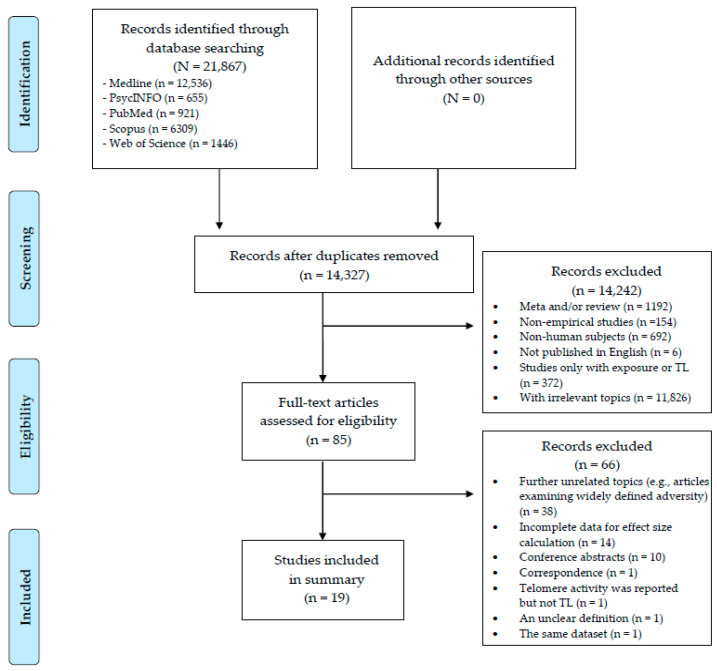
Flow diagram of the study selection process.

A flow chart of the study selection is shown in Figure 1. The systematic research identified 19 studies (N = 18,977). Characteristics of the included studies are summarized in Table 1. Of the 19 selected studies, 15 were of cross-sectional design and 4 were longitudinal. 12 studies involved both male and female participants, and 7 studies had female participants only. Regarding the characteristics of exposure to violence, two reported the co-occurrence of childhood exposure to family violence, and the remaining reported child maltreatment. The Childhood Trauma Questionnaire (CTQ) and the Revised Conflict Tactics Scale were most commonly used by the studies to measure child maltreatment and exposure to IPV, respectively.

For the outcome measures, the majority of the studies assessed TL in blood cells (e.g., leukocytes, peripheral cells) (*n* = 13) and some used buccal swabs or saliva. 16 studies collected data on TL during adulthood, and 17 studies used qPCR to analyze TL.

### 3.2. Synthesis of Effect Sizes

**Figure 2:**
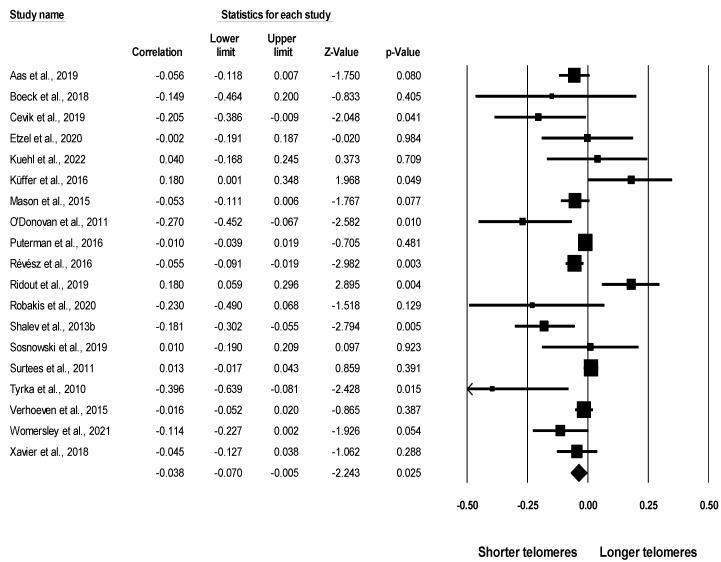
Forest plot of the main association between childhood exposure to family violence and telomere length in the included studies. Note. The squares in the plot show the effect size of the related study. The diamond-shaped rhombus at the bottom of all squares shows the overall effect size obtained from all studies [23,24,25,26,27,28,29,30,31,32,33,34,44,45,46,47,48,49,50].

Figure 2 shows graphically the effect size for each sample. TL had a significant inverse association with childhood exposure to family violence across all 19 of the selected studies (*r* = −0.038, 95% CI [−0.070, −0.005], *p* = 0.025). The heterogeneity test was significant (Q = 52.790, df = 18, *p* < 0.001), suggesting the possibility of heterogeneity among the studies. The I^2^ statistic (I^2^ = 65.902) showed that more than 60% of the heterogeneity could be attributed to variation; thus, we continued to perform the subgroup analyses.

### 3.3. Subgroup Analyses

Table 2 presents the findings of the subgroup analyses, showing that the strength of the association between childhood exposure to family violence and TL was stronger for studies examining co-occurring types of violence (*r* = −0.209) than for those examining a single type (*r* = −0.025) (Q = 8.143 *p* = 0.004). Larger effect sizes were found in smaller sample sizes (*n* < 100) (*r* = −0.132) than in medium (*n* >100 and <1000) (*r* = −0.036) and larger samples (*n* > 1000) (*r* = −0.023) (Q = 3.398, *p* = 0.183). No significant moderating effects were found for gender (Q = 0.008, *p* = 0.931), types of violence measurement (Q = 0.360, *p* = 0.549), source of tissue (Q = 4.396, *p* = 0.111), telomere measurement technique (Q = 0.002, *p* = 0.963), or time of telomere measure (Q = 0.280, *p* = 0.597), etc.

### 3.4. Sensitivity Analysis

The sensitivity analysis showed that removal of the studies did not alter the association.

### 3.5. Publication Bias

As shown in Figure S1, no evidence of publication bias was found in the present meta-analysis. The tests of Egger’s regression and Begg–Mazumdar rank correlation were both insignificant (*p* > 0.05).

## 4. Discussion

This meta-analysis identified 19 articles covering 18,977 individuals and found that childhood exposure to family violence had a significant negative effect on TL (*r* = −0.038, *p* = 0.025). Most previous systematic reviews and meta-analytic studies had found a significant association between widely defined adversity and TL [12,20]. A key difference between the present study and most prior work mentioned above is that they defined adversity as a general and broad conception, for example, loss of a close family member, general trauma, and childhood exposure to family violence. Given that family violence in childhood is prevalent as indicated in Introduction, this global measure of adversity prevents previous work from exploring a pure effect of childhood exposure to family violence. Our current objective to see the specific association between childhood exposure to family violence and TL echoes Pepper et al., (2018)’s idea that it is necessary to explore adversity separately [21]. Understanding this accurate association may provide information on effective and targeted prevention and intervention programs geared toward victims who exposed to family violence during childhood, which not only improves the efficient allocation of resources for services, but also assists the victims with family trauma treatments.

Several possible pathways could explain this finding. First, chronic stress resulting from childhood exposure to family violence increases the activation of the hypothalamic-pituitary-adrenocortical (HPA) axis, and especially its end product, cortisol. Vitro experiments have demonstrated a causal relationship between elevated cortisol exposure and telomere erosion [51], which may be explained by the downregulating influences of cortisol on telomerase activity [52]. Indeed, a meta-analysis study supports the evidence of an association between salivary cortisol reactivity and telomere shortening [53]. Second, the telomere-erosion process could be triggered by stressful events through inflammation, because inflammation is related to increased proliferation of immune and hematopoietic stem cells and therefore leads to telomere erosion [54]. Third, increased oxidative stress could also damage telomeres because of the high-guanine-rich content in telomeres [54]. Moreover, if these factors (e.g., inflammation and HPA-axis responses) dysregulate simultaneously, that could lead to a cumulative impact on TL [49]. Fourth, unhealthy lifestyles could partly account for shorter TL. For example, people who are exposed to life adversity are more likely to adopt unhealthy lifestyles as coping mechanisms (e.g., smoking) [55], and any of those could activate the potential dysregulation of the HPA axis, which in turn could result in TL shortening [22,49]. Further research will be required to clarify and untangle the abovementioned explanations.

One likely cause of the small overall effect size in this study is qPCR measurement error. TL is commonly measured using the qPCR-based method in the current study, mainly due to its cheapness, quickness, and the small quantities of DNA. However, one obvious limitation of qPCR is that it has higher measurement errors and thus reduces the statistical power to detect associations [56,57]. Some potential sources may lead to the errors, including primer choices, pipetting errors, well position effects, etc. Controlling for those causes might greatly help to minimize measurement errors [56].

Our moderator analyses of the moderating effect exerted by co-occurrence of childhood exposure to family violence showed that co-occurring violence had a significantly larger effect size than non-co-occurrence events did, which is consistent with previous findings that telomeres tend to be shorter among individuals reporting greater life adversity [27]. Meanwhile, additional violence in a family has been shown to exert negative effects on health outcomes [58]. For example, a meta-analytic study with 99,956 participants found that family polyvictimization (e.g., child maltreatment, elder abuse against grandparents, and in-law abuse) was significantly associated with depression and post-traumatic stress disorder [40]. Future studies are needed to further explore and confirm those findings.

We did not find a significant difference between studies involving both genders and those involving females only. The literature has inconsistent findings on gender differences in TL in adults. Males may present with shorter telomeres than females, likely because of complex hormonal processes [32,59]. Testosterone is found to increase the susceptibility to oxidative stress [60], which might increase telomere attrition in males [32]. Longer TL in females might be due to their higher estrogen levels, which could activate telomerase and protect telomeres from erosion [59]. Another reason for shorter TL in males might be because males may be more biologically vulnerable to stressful events (e.g., childhood maltreatment) than females [32]. However, Hunt et al. (2008) did not find a gender effect on TL in adult participants [61]. In the present study, we did not have the raw data to verify those notions. More comprehensive knowledge about gender-specific differences could guide clinical practitioners to provide specific intervention for targeted gender groups.

In checking for a moderating effect from different sample sizes, we found that the studies with smaller sample sizes showed a larger effect size––data that should be interpreted with caution. A review found that findings of larger samples were less conclusive compared to findings of smaller samples [12]. The larger-sample studies may have been able to control for more covariates that are always inter-correlated in larger models and thus to cover up the direct impact.

Studies grouped by the telomere measurement method (qPCR vs. other techniques) were not significantly different in the present study. The literature has conflicting results on the telomere measurement technique [7,20,21]: the qPCR showed a larger effect size than the Southern Blot [20] or other techniques combined did [7], whereas Pepper et al. (2018) found that the Southern Blot did not differ significantly from the qPCR [21]. In addition, in our analysis the different types of tissues in which TL measurements were taken showed no significant differences, which is consistent with the findings from Hanssen et al. (2017) [20] and Pepper et al. (2018) [21]. A study found that saliva and leukocyte DNA lengths were highly and significantly correlated (R = 0.72, *p* = 0.002) [62]. Thus, we recommend that future studies collect samples from multiple tissue types and compare them.

## 5. Strengths, Limitations, and Future Research

This meta-analysis has several strengths. As already mentioned, most previous review studies conceptualized childhood exposure to family violence within a broad definition of adversity, rendering them unable to provide information delineating the relationship between childhood exposure to family violence and TL. The present study offers quantitative findings on this association and on how the strength of this association is affected by moderators. We believe that this is the first meta-analytic study to examine the association between childhood exposure to family violence and TL.

The findings of this meta-analysis advance knowledge about the association between childhood exposure to family violence and TL, but the study findings need to be interpreted with the following caveats. First, we only included cross-sectional data, making it impossible to investigate causality between childhood exposure to family violence and TL. Four articles provided longitudinal data of the effect of childhood exposure to family violence on TL over time, but we chose only to include their baseline data in the analysis because it was difficult to estimate accurately on the basis of only four sets of longitudinal data with different follow-up intervals. Second, our number of included studies was small. Although we tried hard to contact authors of the studies that did not provide sufficient data for calculating their effect sizes, we could not reach some of those authors and that omission limited our ability to fully assess the association. Third, we cannot separate the data of different types of violence to see the specific effects of various forms of family violence as no relative data were available. This is an important area for future studies with more comprehensive data to explore. Furthermore, most studies included in this analysis were from countries in North America and Europe, and the generalizability of those findings to other cultures is uncertain. Finally, this research was limited to studies published in English, thus opening the possibility of cultural bias.

## 6. Implications

The reverse relationships between childhood exposure to family violence and TL make it highly important for practitioners and health professionals to screen for violence in the family, and especially to look for the co-occurrence of multiple types of violence and then to provide timely trauma-informed interventions. Policymakers should consider childhood exposure to family violence to be a risk factor for biological issues (e.g., shortened TL) and should prioritize prevention and intervention for individuals who are exposed to family violence during childhood.

TL throughout life is determined by the interaction between endogenous (genetic) and external (nongenetic) factors [63]. For the external factors, in addition to reducing stressful events (e.g., childhood exposure to family violence as mentioned above), future studies are suggested to explore the positive impacts of resiliency factors on potentially reversing TL. Specifically, at the personal level, maintaining a healthy lifestyle is one of the most studied aspects of protecting TL as indicated in the Discussion. A meta-analysis showed that TL was shorter in ever smokers compared to never smokers. Among ever smokers, current smokers had shorter mean telomeres than former smokers [64]. More recent meta-analyses found that other healthy lifestyles, such as fewer sedentary activities and optimal sleep habits [65], and a greater number of hours of meditation [66], were associated with a greater impact on telomere biology. Future prospective and well-powered intervention trials with standardized protocols and objective measures are needed to examine how these protective factors impact TL [65]. Furthermore, building psychological resilience is of great importance to protect telomeres. A meta-analysis indicated that greater optimism was associated with longer telomeres (a small, weighted effect size, r = 0.06, *p* = 0.02) [67] A community study found that emotional regulation and self-control moderated the association between stress and aging [68]. At the social level, a negative perception of neighborhood context [69] and experiencing discrimination (which may interact with other variables, such as acculturation) [70] may contribute to shortening telomeres. Remarkedly, moderate or high social support could reduce the negative impact of discrimination on TL [71]. Therefore, future work intervening in these modifiable factors is suggested to determine if they are protective against the effects of stress on epigenetic age acceleration. Finally, epigenetics modification may also affect TL and telomere structure [72]. However, these processes are complex, and more studies are needed to understand better the connection between telomeres, epigenetics and aging [73].

Because longitudinal studies are scarce, future research on childhood exposure to family violence and health should include well-designed longitudinal studies from diverse cultural backgrounds to further confirm the current findings. Furthermore, because the relationship between co-occurring types of childhood exposure to family violence and TL was found to be stronger than that between TL and a single type of violence, future studies should focus on the added negative impacts of family polyvictimization. To complete the picture, future studies should look closely at the mediating role of TL in the relationship between co-occurring childhood exposure to family violence and health-related consequences. Additional research is also needed on other potential moderators (e.g., gender) and mediators of the association between childhood exposure to family violence and TL.

## 7. Conclusions

This study contributes to the current knowledge by documenting that childhood exposure to family violence is negatively associated with TL, thus further indicating that such violence impairs victims’ biological health. In addition, the strength of this association was stronger in studies examining the co-occurrence of childhood exposure to family violence than in those examining one type.

## Figures and Tables

**Table 1:** Characteristics of measures used in the included studies.

Study Characteristics					Participants’ Characteristics			Exposure of Violence				Telomere Measurement		
Authors (Year)	Country	Sample	Study Design	Sample Size	Mean Age (s.d.)	Female (%)	Education	Types of Violence	Specific Age Range of Violence Happened	Measures to Detect Violence	Mode of Reporting	TL Cell Type	Period of TL Measurement	TL Assay Type
Aas et al., 2019 [28]	Norway	The participants were recruited from psychiatric units (outpatient and inpatient) of four major hospitals	CS	Schizophrenia (SZ) = 373, bipolar disorder (BD) = 249, healthy (HC) = 402	SZ: 29.1 (9.3), BD: 31.8 (11.3), HC: 31.4 (7.6)	SZ: 41%, BD: 58%, HC: 43%	NA	Sexual abuse, physical abuse, and emotional abuse	Not reported	Childhood Trauma Questionnaire (CTQ)	Self-report	Blood	Adulthood	qPCR
Boeck et al. 2018 [30]	Germany	Women giving birth in the maternity ward of the University Hospital Ulm were invited to participate in the study	CS	30	CM− = 31.5 (5.56), CM+ = 30.9 (6.4)	All female	University: CM− = 60.0%, CM+ = 33.3%	Physical/emotional/sexual abuse and physical/emotional neglect	≤18	CTQ	Interview	Blood	Adulthood	qFISH
Çevik et al., 2019 [44]	Turkey	Participants of a large gene-environment interaction study: European Network of National Schizophrenia Networks Studying Gene-Environment Interactions	CS	Schizophrenia (SCZ) = 100	SCZ = 31.69 (8.01)	SCZ = 32%	≥university: SCZ = 15%	Physical abuse, psychological abuse, and sexual abuse	≤17	Childhood Experience of Care and Abuse- Interview (CECA-Interview)	Interview	Blood	Adulthood	qPCR
Etzel et al., 2020 [45]	The United States	Female subjects with substantiated sexual abuse were referred to the study by Child Protective Services (CPS) agencies. Control subjects were recruited from the same communities as the childhood sexual abuse (CSA)-exposed participants through local advertisements	CS	108	At DNA collection: 36.3 (3.3)	All female	16.5 (1.9)	Sexual abuse	6–16	Substantiated by Child Protective Services	Referred by Child Protective Services	Buccal	Adulthood	qPCR
Kuehl et al., 2022 [46]	Germany	Patients and healthy participants were recruited from the specialized affective disorder unit and by public postings	CS	90	MDD+/ACE+ (N = 23): 38.1 (11.4); MDD+/ACE− (N = 24): 32.7 (11.5); MDD−/ACE+ (N = 22): 34.7 (10.7); MDD−/ACE− (N = 21): 36.1 (11.4)	64.44%	MDD+/ACE+: 11.3 (1.6); MDD+/ACE−: 12.0 (1.4); MDD−/ACE+: 11.8 (1.4); MDD−/ACE−: 12.1 (1.3)	Physical or sexual abuse	≤18	CTQ	Self-report	Blood	Adulthood	qPCR
Küffer et al., 2016 [25]	Germany	Participants were recruited via advertisements in local and national newspapers and magazines, and via specific indentured child laborers’ societies and associations	CS	Former indentured child laborers = 62, healthy controls = 58	Former indentured child laborers = 76.19 (6.18), healthy controls = 71.85 (5.97)	Former indentured child laborers = 43.55%, Controls = 44.11%	Former indentured child laborers = 10.45 (2.16), controls = 13.35 (3.57)	Emotional/physical/sexual abuse and emotional/physical neglect	Not reported	Childhood Trauma Questionnaire − Short Form (CTQ-SF)	Self-report	Buccal	Adulthood	qPCR
Mason et al., 2015 [26]	The United States	The Nurses’ Health Study II (NHSII) follows 116,430 female registered nurses	CS	1135	Between the ages of 25 and 42	All female	NA	Physical and sexual abuse	≤17	Revised Conflict Tactics Scale Sexual experiences survey	Self-report	Blood	Adulthood	qPCR
O’Donovan et al., 2011 [47]	The United States	Participants were recruited through ads and flyers distributed in the community, as well as through relevant local clinics for the PTSD sample	CS	PTSD = 43, controls = 47	PTSD = 30.60 (6.63), controls = 30.68 (8.19)	PTSD = 47%, controls = 56%	PTSD: female (*n* = 20) = 15.2 (2.1), male (*n* = 22) = 14.4 (2.3); Controls: female (*n* = 25) = 15.4 (2.0), male (*n* = 21) = 15.5 (2.1)	Physically harmed, physical neglect, family violence, physical abuse, forced sexual touch, or forced sexual intercourse	≤14	Life Stressor Checklist (LSC)	Interview	Blood	Adulthood	qPCR
Puterman et al., 2016 [27]	The United States	The participants were from an ongoing longitudinal, nationally representative sample of >26,000 US residents over 50 years of age and their spouses	CS	4598	<60: 25.7%	55.90%	College and above: 25.4% (*n* = 4597)	Physically abuse	≤18	Major childhood adversity items were asked across the survey modules	Self-report	Saliva	Adulthood	qPCR
Révész et al., 2016 [29]	The Netherlands	Respondents were recruited from community, primary care, and specialized mental health care settings	L	Baseline = 2936, 6-year follow-up = 1860	Baseline = 41.81 (13.07)	66.40%	12.15 (3.27)	Emotional neglect, psychological abuse, physical abuse or sexual abuse	≤16	Childhood Trauma Interview (CTI)	Interview	Blood	Adulthood	qPCR
Ridout et al., 2019 [24]	The United States	Children with maltreatment were identified from the local child welfare agency or an emergency maltreatment assessment service via recorded review. Families without maltreatment were recruited at a pediatric medical clinic during a well-child visit or at childcare centers	L	No maltreatment = 123, maltreated = 133	No maltreatment = 50.1 (9.0) (months), maltreated = 51.9 (8.8) (months)	No maltreatment = 51.2%, maltreated = 53.4%	NA	Physical/sexual abuse, physical neglect/failure to provide, physical neglect/lack of supervision, emotional maltreatment	Not reported	System for Coding Subtype and Severity of Maltreatment in Child Protective Records	Official record	Saliva	Childhood	qPCR
Robakis et al., 2020 [48]	The United States	The clinical-women sample was recruited from local obstetric clinics, community postings, and the Stanford University reproductive psychiatry clinic. The epigenetic sample was recruited in part from the clinical sample population and in part from a second study with equivalent recruitment criteria and follow-up procedures	CS	Epigenetic sample = 54, clinical sample = 124	Epigenetic sample: 32.33 (4.40), clinical sample: 32.31 (4.79)	All female	Above bachelor: epigenetic sample = 87.04%, clinical sample = 82.26%	Physical/emotional/sexual abuse and physical/emotional neglect	Not reported	CTQ	Self-report	Buccal	Adulthood	qPCR
Shalev et al., 2013b [23]	United Kingdom	The sample was drawn from a larger birth register of twins born in England and Wales in 1994–1995	L	236	Baseline = age 5	49%	NA	Domestic violence and physical maltreatment	5–10	Conflict Tactics Scale Physical maltreatment	Interview mothers (or the primary caregiver)	Buccal	Childhood	qPCR
Sosnowski et al., 2019 [33]	The United States	The present study group consisted of a subset of female–female (FF) monozygotic (MZ) twins who participated in the population-based Virginia Adult Twin Study for Psychiatric and Substance Use Disorders	CS	97	52.74 (8.55)	All female	14.67 (2.14)	Childhood sexual abuse	≤16	A single item from an adapted version of a previously developed measure	Self-report	Blood	Adulthood	MMqPCR
Surtees et al., 2011 [34]	United Kingdom	As virtually 100% of people in the United Kingdom are registered with general practitioners through the National Health Service, the age–sex registers form a population-based sampling frame	CS	4441	62 years (range 41 and 80)	All female	NA	Physical abuse	≤17	the Health and Life Experiences Questionnaire (HLEQ)	Self-report	Blood	Adulthood	qPCR
Tyrka et al., 2010 [31]	The United States	Subjects were recruited via advertisements in the community for a larger study of stress reactivity and psychiatric symptoms	CS	No-maltreatment = 21, maltreatment = 10	26.9 (10.1)	No maltreatment = 67%, Maltreated = 80%	Above College: No maltreatment = 61.9%; Maltreated = 40%	Physical/sexual/emotional abuse and physical/emotional neglect	Not reported	CTQ	Self-report	Blood	Adulthood	qPCR
Verhoeven et al., 2015 [49]	The Netherlands	Participants were assessed during a 4-hour clinic visit	CS	2936	41.8 (13.1)	66.4%	12.2 (3.3)	Emotional neglect, psychological abuse, physical abuse, or sexual abuse	≤16	Childhood Trauma Interview (CTI)	Interview	Blood	Adulthood	qPCR
Womersley et al., 2021 [50]	South Africa	Women were recruited over 8 years (2008–2015) from community health care facilities in and around Cape Town, South Africa	L	286	Baseline = HIV−ve: 28.58 (8.36); HIV + ve: 33.11 (6.90)	All female	HIV−ve: 10.83 (1.45); HIV + ve: 10.12 (1.68)	physical, emotional and sexual abuse, as well as physical and emotional neglect	≤18	CTQ	Self-report	Blood	Adulthood	qPCR
Xavier et al., 2018 [32]	Brazil	Participants from a large prospective community school-based study	CS	561	10.19 (1.91)	45.10%	NA	Physical abuse, neglect, emotional maltreatment, and sexual abuse	Not reported	Four questions regarding the history of adverse environment and trauma	Self and the parent-report	Blood	Childhood	qPCR

Note. qFISH = quantitative fluorescent in situ hybridization. qPCR = quantitative polymerase chain reaction. MMqPCR = a monochrome multiplex qPCR technique. CM = child maltreatment. MDD = Major depressive disorder. ACE = Adverse childhood experiences. CS = cross-sectional design. L = longitudinal. TL = telomere length. NA = not applicable.

**Table 2:** Categorical moderator analysis.

Moderator	Random Effect Size Estimate				Heterogeneity Analysis			
	k	*r*	95% CI	*p*	Q	df	*p*	I^2^
Gender, Q (1) = 0.008, *p* = 0.931								
Both genders	12	−0.038	[−0.080, 0.005]	0.082	40.897	11	<0.001	73.103
Females only	7	−0.041	[−0.103, 0.021]	0.194	10.188	6	0.117	41.110
Co-occurrence of violence ^a^, Q (1) = 8.143, *p* = 0.004								
Non-co-occurrence (single type of occurrence)	17	−0.025	[−0.056, 0.005]	0.106	40.894	16	0.001	60.874
Co-occurrence	2	−0.209	[−0.325, −0.087]	0.001	0.558	1	0.455	<0.001
Violence measurement, Q (1) = 0.360, *p* = 0.549								
Self-report	10	−0.029	[−0.075, 0.018]	0.225	21.790	9	0.010	58.696
Others	9	−0.050	[−0.100, 0.001]	0.054	27.722	8	0.001	71.143
Source of tissue, Q (2) = 4.396, *p* = 0.111								
Blood (e.g., Leukocytes, peripheral cells)	13	−0.056	[−0.098, −0.014]	0.009	28.300	12	0.005	57.598
Buccal swabs	4	−0.054	[−0.158, 0.052]	0.318	12.000	3	0.007	74.999
Saliva	2	0.050	[−0.041, 0.139]	0.283	8.875	1	0.003	88.733
Telomere measurement technique, Q (1) = 0.002, *p* = 0.963								
qPCR	17	−0.038	[−0.072, −0.004]	0.027	52.187	16	<0.001	69.341
Other techniques	2	−0.033	[−0.220, 0.155]	0.730	0.595	1	0.441	<0.001
Time of telomere measure, Q (1) = 0.280, *p* = 0.597								
Adulthood	16	−0.042	[−0.078, −0.005]	0.025	35.927	15	0.002	58.249
Childhood	3	−0.017	[−0.101, 0.066]	0.685	16.861	2	<0.001	88.138
Whether covariates controlled ^b^, Q (1) = 0.618, *p* = 0.432								
Yes	4	−0.015	[−0.084, 0.053]	0.663	17.370	3	0.001	82.729
No	15	−0.047	[−0.088, −0.006]	0.023	35.366	14	0.001	60.414
Sample size, Q (2) = 3.398, *p* = 0.183								
Large (>1000)	5	−0.023	[−0.067, 0.021]	0.311	9.806	4	0.044	59.209
Medium (100–1000)	8	−0.036	[−0.090, 0.018]	0.189	27.718	7	<0.001	74.746
Small (<100)	6	−0.132	[−0.237, −0.024]	0.017	9.505	5	0.091	47.397

Note. *r* = correlation. CI = confidence interval. ^a^ O’Donovan et al., (2011) and Shalev et al., (2013b) provided the data of co-occurrence of violence. ^b^ Aas et al., (2019) and O’Donovan et al., (2011) adjusted for age and gender. Ridout et al., (2019) adjusted for age and ethnicity. Verhoeven et al., (2015) adjusted for age.

## Data Availability

Data have been included in the articles.

## Supplementary Materials

The following supporting information can be downloaded at: https://www.mdpi.com/article/10.3390/ijerph191912151/s1, Figure S1: Funnel plot of standard errors by Fisher’s Z transformation; Table S1: Quality assessments of the included studies.
