# Supplementary material for: Association between Childhood Exposure to Family Violence and Telomere Length: A Meta-Analysis
Source: Int J Environ Res Public Health. 2022 Sep 26;19(19):12151. doi: 10.3390/ijerph191912151 (PMC9566190; doi:10.3390/ijerph191912151)
Supplement: Supplementary file 1 [file ijerph-19-12151-s001.zip › ijerph-1889612-supplementary done.pdf]

Table S1. Quality assessments of the included studies

	Criteria								Score
**Authors (Year)**	**1**	**2**	**3**	**4**	**5**	**6**	**7**	**8**	
Aas et al., 2019	√	√	√	√	√	√	√	√	8
Boeck at al., 2018	√	√	√	√	√	√	√	√	8
Çevik at al., 2019	√	√	√	√	√	√	√	√	8
Etzel at al., 2020	√	√	√	√	√	√	√	√	8
Kuehl at al., 2022	√	√	√	√	√	√	√	√	8
Küffer et al., 2016	√	√	√	√	√	√	√	√	8
Mason at al., 2015	√	√	√	√	√	√	X	√	7
O'Donovan et al., 2011	√	√	√	√	√	√	√	√	8
Puterman et al., 2016	√	√	√	√	√	√	X	√	7
Révész et al., 2016	√	√	√	√	√	√	√	√	8
Ridout et al., 2019	√	√	√	√	√	√	√	√	8
Robakis et al., 2020	√	√	√	√	√	√	√	√	8
Shalev et al., 2013b	√	√	√	√	√	√	√	√	8
Sosnowski et al., 2019	√	√	√	√	√	√	X	√	7
Surtees et al., 2011	√	√	√	√	√	√	X	√	7
Tyrka et al., 2010	√	√	√	√	√	√	√	√	8
Verhoeven et al., 2015	√	√	√	√	√	√	√	√	8
Womersley et al., 2021	√	√	√	√	√	√	√	√	8
Xavier et al., 2018	√	√	√	√	√	√	√	√	8

Note.
Criterion 1: Did the study clearly report its design (e.g., cross-sectional, longitudinal)?
Criterion 2: Did the study report its sampling procedure?
Criterion 3: Did the study clearly define exposure measures?
Criterion 4: Did the study report the age of the exposure?
Criterion 5: Did the study clearly define the measurement of telomeres?
Criterion 6: Did the study clearly describe the sample characteristics?
Criterion 7: Were inclusion and/or exclusion criteria stated?
Criterion 8: Did the study provide sufficient data to analyze?

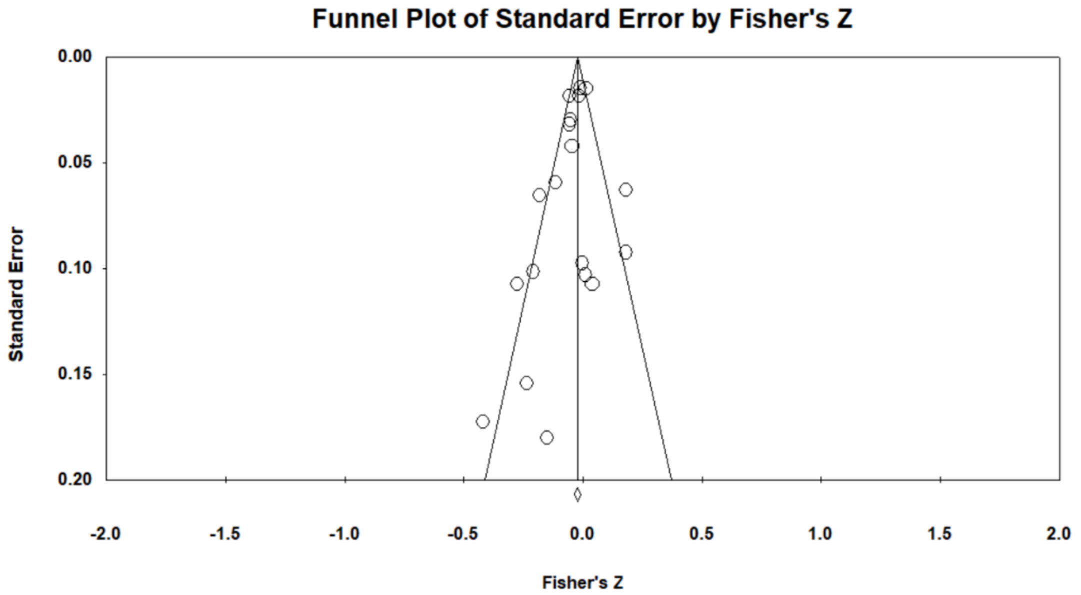

Figure S1. Funnel plot of standard errors by Fisher's *Z* transformation.
